# Cuba—U.S. scientific collaboration: Beyond the embargo

**DOI:** 10.1371/journal.pone.0255106

**Published:** 2021-07-22

**Authors:** Guillermo Armando Ronda-Pupo

**Affiliations:** 1 Coordinación General de Desarrollo Estratégico y Calidad, Universidad Católica del Norte, Antofagasta, Chile; 2 Departamento de Administración y Economía, Universidad de la Frontera, Temuco, Chile; University of Central Florida, UNITED STATES

## Abstract

Cuba and the U.S. have the oldest Academies of Sciences outside Europe. Both countries have a long history of scientific collaboration that dates to the 1800s. Both scientific communities also share geographical proximity and common scientific research interests mainly in Biotechnology, Meteorology, and Public Health research. Despite these facts, scientists from both nations face serious barriers to cooperation raised by the U.S. embargo established in 1961 that prohibits exchanges with Cuba. The study aims to analyze the effects of U.S. policy on scientific collaboration with Cuban scientific institutions. The results of the bibliometric analysis of Cuba-U.S. joint publications in the Web of Science, and Scopus databases between 1980 to 2020 indicate sustained growth of scientific collaboration between scientists of both nations over the past forty years. The results also show that after the 1980 Smithsonian Institution and the Cuba’s Academy of Sciences agreement significantly increased scientific collaboration between U.S. scientists with their Cuban peers. President Barack Obama’s approach to normalizing the U.S. Cuba relations in 2015 enhanced Cuban scientific production with U.S. scientists by exceeding the number of collaborative papers published during any preceding U.S. Presidential administration. By 2020, Cuba had expanded its scientific links to 80% of the countries in the world. Cuban and U.S. scientists converted from adversaries into partners, showing that science is an effective diplomatic channel. A particularly important question for the future is how robust is the collaboration system in the face of greater political restrictions?

## 1 Introduction

Cuba and the U.S. have a long history of scientific collaboration that dates to the 1800s. Cuban naturalist Felipe Poey deposited many of his specimens, results of his studies on fish species in the waters around Cuba, in such U.S. institutions as Smithsonian and Harvard University [1]. In the late 19th Century the Cuban scientist Carlos Finlay’s scientific collaboration with Jesse Lazear of Johns Hopkins University corroborating Finlay’s theories presented 20 years earlier on mosquitoes as the vector for yellow fever transmission [2]. Those actions paved the way for closer collaboration between scientists in the United States and Cuba.

The Cuban science system shows important achievements, particularly in human health care and biotechnology [3]. https://www.nejm.org/action/showMediaPlayer?doi=10.1056%2FNEJMdo002385&aid=10.1056%2FNEJMp1608859&area=. Cuba has lower rates of infant mortality than a number of American communities [4]. The Cuban health care system is recognized for its undeniable successes including its emphasis on primary care and prevention [5, 6]. Of pregnant women, 95% receive prenatal care, which reduced the infant mortality rate to < 5 per 1000 births [6, 7]. Vaccination rates in Cuba are among the highest in the world. The life expectancy of 77 and 81 years of age in men and women, respectively, is nearly equal to that in the United States [6, 7]. Recently, Cuba became the first Latin American country with four COVID–19 vaccines in the third experimentation stage [8].

Cuba–U.S. collaboration is essential to enhance research on their mutual scientific interests [9]. Both scientific communities share an interest in studying tropical cyclone forecasting [10, 11], animal and plant health, neuroscience [12] biotechnology [13, 14], bioethics [15], cancer, public health, environmental sciences including joint monitoring of coral reef ecosystems [2], infectious diseases Dengue and Chikungunya, agriculture and immunology [16], gastroenterology [7] marine sciences, informal science education [4, 17], tuberculosis eradication [18], and chemical research [19–21].

Despite these joint scientific interests, after the 1959 Cuban revolution, the political divide between the U.S. and Cuba started to grow, leading to severing of diplomatic relations [2]. During the past 60 years, the two countries’ relationship has been subject to complex economic, political, and social forces [2]. In 1961 the U.S. raised the embargo on exchanges with Cuba that continues today. The economic embargo prohibits all exports to Cuba except for some food and medicine. It also forbids the spending of money in Cuba by U.S. citizens without a license from the U.S. Department of the Treasury effectively blocking any U.S. research funding from reaching Cuban scientists and research institutions [12, 16] and restraining the scientific collaboration in many fields [22].

In the early 1980s, the U.S.–Cuba collaboration began to grow with the agreement between the U.S. Smithsonian Institution and the Cuba’s Academy of Sciences (CAS). In the 1990’s agreement between New York Botanical Garden and the CAS was also initiated [23, 24]. In 1999, the Clinton administration announced a new policy to expand people–to–people (p2p) contacts—such as scientific exchanges—between the United States and Cuba and established a general license in effect for people in certain categories, including diplomats, journalists, and academics. This means that U.S. university scientists can travel to Cuba, if they work on academic pursuits during the visit, with the intention to produce scientific publications. It is important to highlight that the existence of this general license is not widely known [1, 2], and obtaining a visa to visit Cuba is a cumbersome and long process [7].

In a 2009 visit eight U.S. science leaders, led by AAAS President Peter Agre, visited Cuba aiming to foster cooperative projects to address a range of shared U.S. Cuba scientific interests [4]. In 2015 the Obama administration encouraged the reestablishment of diplomatic ties between the U.S. and Cuba, thereby promoting the expansion of scientific collaboration by general authorization for joint commercial and non-commercial medical research [2]. These significant steps toward normalization of Cuba–U.S. relations by the Obama administration were partially reversed in 2017 when Donald Trump’s administration revised U.S. policies toward Cuba (available at https://home.treasury.gov/policy-issues/financial-sanctions/sanctions-programs-and-country-information/cuba-sanctions). These latter changes dissuaded both sides from pursuing joint research, restricting scientific collaboration [2]. With the election of Democrat Joe Biden, there was hope that flexible of scientific collaboration between the U.S. and Cuba was back [25], but frequent large policy shifts make research planning uncertain and act as a deterrent to collaboration even in less restrictive times.

The Cuban scientific production in the Clarivate Analytics Web of Science (WoS), and Elsevier Scopus increased exponentially in the last 40 years. The internationalization, strategic alliances, and the geographical diversification of its international collaboration network have allowed Cuba to continue to develop its science ecosystem [23]. Beyond the legal restrictions, Cuban and U.S. scientists have managed to continue to share and build bridges of cooperation by advancing joint research projects. The scientific engagement and knowledge exchange between the scientific communities of both nations acts to moderate political barriers [2]. Thus, it is interesting to shed light on the results of that winding scientific relationship by answering the following questions:

RQ 1: Has U.S.–Cuba scientific collaboration favored the growth of the Cuban international scientific collaborative network?
RQ 2: Which U.S. scientific approach/agreement, Smithsonian 1980, New York Botanical Garden 1990, Clinton’s p2p 1999 policy, AAAS 2009, or Obama 2015 policy, have been most effective in enhancing the Cuba–U.S. joint scientific production as reflected in the WoS and Scopus?
RQ 3: What are the necessary conditions for Cuba to enhance scientific collaboration with the U.S?

## 2 Materials and methods

### 2.1 The data

The data consists of joint publications of Cuban–U.S. scientists between 1980 and 2020, inclusive in the publication databases WoS and Scopus. The data was retrieved on April 30, 2021. We used the query Advance search in WoS Core Collection CU = (Cuba) and PY = 1980–2020 and DT = (article or review or proceedings paper), Citation Indexes: Science Citation Index Expanded, Social Science Citation Index, and Arts & Humanities Citation Index. We did not include the Emerging Sources Science Citation Index because it only indexed documents from 2015 onwards, and may introduce bias favoring the Obama 2015 stage. We analyzed the results and filtered the data using the label Country/region to select the Cuban papers that were published with the participation of at least one U.S. researcher. This procedure was also used to retrieve the overall Cuban productivity excluding the U.S. collaboration.

To retrieve the productivity from the Scopus database we search using Affiliation Country = Cuba, and filtered the results by Year: (1980–2020), Document Type. (Article, Conference Paper, and Review), and Country/Territory (United States) to select the Cuban scientific productivity in collaboration with the U.S, also the Cuban scientific productivity without the participation of the U.S. In both queries we retrieved two datasets. One dataset that includes only the Cuba–U.S. joint publications. The second retrieved the overall Cuban scientific production excluding the articles with the participation of U.S.

The results from the WoS, and Scopus databases were combined using the R program bibliometrix [26], an open-source tool for quantitative research in Scientometrics. This program brings many advantages, it removes duplicates, and it is possible to run several bibliometric based analyses.

### 2.2 Time frame

The dataset covers the Cuban publications in the WoS, and Scopus from 1980 to 2020, inclusive. We include the past 41 years for several reasons. From 1960 to 1980, the U.S.–Cuba scientific collaboration was restricted. The Cuban science system collaborated mainly with socialist countries from Eastern Europe [23]. The 41-year time span is a significant time frame because it includes the Cuban scientific output ten years before the collapse of the USSR, and it spans the five main milestones in the Cuba–U.S. scientific collaboration. For the analysis of the historical evolution of the Cuban international scientific collaborative network (Fig 3), we also retrieved the information 1900–1979 using the same queries in WoS, and Scopus.

### 2.3 The variables

#### 2.3.1 Scientific production

One accepted measure of scientific production is defined as the number of peer-reviewed documents (articles, proceedings papers, and reviews) published by a science system in scholarly journals. For the present study, the Cuban scientific production is the total of peer-reviewed papers published by Cuban scientists in the WoS, and Scopus. We used two measures: 1) Cuban overall scientific productivity excluding the papers with the participation of the U.S., 2) The Cuba–U.S. joint scientific production. Previous studies analyzed the Cuban scientific output in scholarly journals [27], the productivity in biotechnology [28], the Cuban productivity of publications and patents [3]. The results of the study will shed light on the Cuban scientific productivity in collaboration with U.S.

#### 2.3.2 Scientific collaboration

Collaboration in research is defined as “the working together of researchers to achieve the common goal of producing new scientific knowledge” [29]. Beaver and Rosen pioneered the study of scientific collaboration in bibliometrics [30, 31]. Bibliometric studies use co-authorship to analyze scientific collaboration [32, 33]. Katz and Martin [29] claim that co-authorship is the accurate method to study scientific collaboration. First, it is invariant and verifiable [29]. Once a co-authored paper is published it will never change. Second, it is a relatively inexpensive and practical method for quantifying collaboration [29]. Third, the size of the sample that is possible to analyze using co-authorships can be very large and the results should be therefore more significant than those from case studies [29].

The number of international addresses in co-authored peer-reviewed papers grew exponentially in the past 20 years [34]. [35] found that the co-authors’ nationality is one of the moderating variables in the relationship between collaboration and productivity. Scientific collaboration enhances the quality of scientific research, improves the efficiency and effectiveness of that research, and is increasingly necessary, as the scale of both budgets and research challenges grow [36]. Fostering collaboration with more developed nations is a driver to increase productivity. Emerging economy countries use international scientific collaboration as a driver to foster their scientific productivity [37]. [33] found that scientific collaboration is also a driver to increase the number of citations of papers. [38] confirmed that the international collaborative papers receive more citations than domestic ones. [23] reported that the Cuban science system fostered scientific productivity and impact on natural sciences through international scientific collaboration.

#### 2.3.3 U.S.-Cuba scientific collaboration

The Cuba–U.S. scientific collaboration between 1960 and 2020 has been difficult. The scientific communities of both countries have made efforts to collaborate in projects of joint scientific interest. The scientific collaboration between the U.S. and Cuba has attracted interest [7, 12, 21, 39]. The literature on this topic is split into two main research streams. The first, analyzed opportunities and joint interests [7, 18, 20, 21, 25, 40]. The second explored the threads and weaknesses to overcome the political restrictions to a normal U.S–Cuba scientific collaboration and the advantages that scientific collaboration would bring to both countries [1, 12, 14, 39]. The dynamics of the evolution of the scientific collaborative activity between the Cuban and the U.S. science systems can be summarized in five important historical moments:

1. 1961. The freeze of the relationship after the 1959 Cuban revolution.
2. 1980. The agreement of the Smithsonian Institution and the Cuba’s Academy of Sciences.
3. 1990. The agreement between the New York Botanical Garden and the Cuba’s Academy of Sciences.
4. 1999. Clinton’s approach through the people-to-people policy (p2p) [1].
5. 2009. The visit of U.S. science leaders, led by AAAS President Peter Agre to the Cuban Academy of Sciences [4].
6. 2015. President Obama encouraged a reestablishment of diplomatic ties between the U.S. and Cuba [2].

It is important to elucidate whether the U.S. scientific approaches to the Cuban scientific actors enhanced Cuba-U.S. joint scientific productivity. Also, if the improvement of Cuba–U.S. scientific links advanced the growth of the size of the Cuban international scientific collaborative network.

### 2.4 Statistical and mathematical procedures

**RQ1:** To answer the research question 1, we used Kruskall Wallis (K-W) nonparametric tests to compare the effects of the U.S. approaches with the Cuban scientific community on Cuba–U.S. joint scientific production. As there is no post hoc test built into the K–W test, to check for mean ranks differences of the pairs of U.S. Cuba agreements, contacts or policies on Cuba–U.S. joint productivity we used a Bonferonni corrected $p\approx 0.0125\left(\frac{0.05}{4}\right)$ to indicate statistical significance [41].

**RQ2:** To answer research question 2, we use the power-law regression model using Eq 1 [23, 42, 43], where k and *α* are constants, ℴ is the standard error of the estimate. *α* is the scaling exponent (slope of the log-log regression line) using the Marquardt-Levenberg algorithm [44], *S* is Cuba–U.S. scientific collaborative network, *C* is the Cuban overall international scientific collaborative network excluding the U.S. The statistical assumptions of the test are the source population normality (Shapiro-Wilk), the constant variance of the dependent variable in the source population regardless of the value of the independent variable, and the independence of residuals (Durbin-Watson Statistic). To evaluate the goodness of fit of the model, we used the Predicted Residual Error Sum of Squares (PRESS).

A scaling or power-law relation exist between two variables, *x* and *y*, if they are correlated by a power-law given by equation *y* = *ax*^*b*^, where *b* is the scaling factor and *a* is a constant [43, 45]. [45, 46] proved that any pair of coupled exponential or linear processes will exhibit a power-law relation with exponent *n* and intercept *s* that is predictable from the exponents and intercepts of the individual exponential processes [45, 47].


$$S=k{C^{\alpha \pm \sigma }}^{}$$ (1)

For practical interpretation of results, if the exponent *α* = 1, the correlation is linear, both variables grow at the same rate. If *α*<1, there is a sublinear scaling correlation suggesting a cumulative disadvantage [48]. The *x* variable is growing faster than *y*. For the present study, it would suggest that the growth of the Cuban overall international scientific collaborative network (*C*) does not influence the grow of Cuba–U.S. scientific collaboration (*S*). Conversely, if *α*>1, there is a superlinear correlation [23, 38, 42, 45, 49]. It would suggest the presence of a cumulative advantage or preferential attachment [23, 38, 42, 45, 50], then *y* variable is growing faster than *x*. In our case of study, the growth of the Cuban overall international scientific collaborative network (*C*) enhance the Cuba–U.S. scientific collaboration (*S*).

As any pair of couple exponential processes exhibit a power-law relationship, it is important to ascertain the existence of a true power-law correlation. To this aim we followed [51]. The procedure uses the parameters α (scaling exponent) and *r* (Pearson correlation) from Ordinary Least Squares (OLS) to calculate the scaling correlation through Standardized Major Axis (SMA) see Eq 2 below. If *α*_*SMA*_≈*α*_*OLS*_, the variables are highly correlated. When no scaling correlation exists always *α*_*SMA*_>*α*_*OLS*_.


$$\alpha_{SMA}=\frac{\left|\alpha_{OLS}\right|}{r_{xy}}when\;r_{xy}\neq 0$$ (2)

## 3 Results

### 3.1 Cuba-U.S. joint scientific production

Table 1 shows the Cuba–U.S. joint scientific output for the period 1980–2020 in the WoS, and Scopus. Three fields, Clinical Medicine, Physics & Astronomy, and Biology, account for 64,6% of the overall productivity of Cuban–U.S. scientific collaboration. This result suggests that U.S. collaboration has been significant in the domains of Natural and Health Sciences. This is consistent with the results reported by [52], showing a correlation between scientific production and collaboration levels.

**Table 1:** Cuban scientific output in collaboration with the U.S. according to the field of research January 1, 1980- December 31, 2020.

Field	Productivity	% Field	Mean authorM
**Physics & Astronomy**	729	27.6%	767
**Clinical Medicine**	659	24.9%	26
**Biology**	321	12.1%	7
**Biomedical Research**	185	7.0%	15
**Public Health & Health Services**	163	6.2%	18
**Earth & Environmental Sciences**	101	3.8%	34
**Agriculture, Fisheries & Forestry**	97	3.7%	7
**Chemistry**	94	3.6%	8
**General Science & Technology**	68	2.6%	61
**Enabling & Strategic Technologies**	68	2.6%	7
**Engineering**	35	1.3%	7
**Social Sciences**	31	1.2%	4
**Psychology & Cognitive Sciences**	24	0.9%	18
**Information & Communication Technologies**	20	0.8%	4
**Historical Studies**	14	0.5%	3
**Economics & Business**	9	0.3%	3
**Mathematics & Statistics**	12	0.5%	6
**General Arts, Humanities & Social Sciences**	7	0.3%	2
**Philosophy & Theology**	7	0.3%	6
**Visual & Performing Arts**	2	0.1%	2

Fields are organized according to Science Metrix, available from http://science-metrix.com/en/news/science-metrix-launches-the-second-public-release-of-its-multilingual-journal-classification. The information is based in the WoS, including the SCI-Expanded, SSCI, and A&HCI citation indexes, and Scopus. The Table includes only the number of documents published in Cuba-U.S. cooperation. 154 papers are not included (5%) because the journal does not appear in the Science Metrix classification list.

Given to common disease problems and geographical proximity it is not surprising that clinical medicine is among the highest collaboration rate. It is less obvious that physics and astronomy should also shows a high collaboration rate. Within the field, the subfields nuclear & particle physics, and applied physics account for 80.5% of the U.S.–Cuba collaborative productivity (Table 2). The mean number of authors per publication in this field is 767. The mean of authors per paper in the subfield Nuclear & Particle Physics is 878, Applied Physics 700, and General Physics 826. The results reveal the highly collaborative and international nature of these disciplines. We thus infer that U.S. scientists involved in these projects collaborate indirectly with Cuban scientists without violating the legal restrictions imposed by the U.S. government. In support of this conclusion, we see little collaboration in the related, but less collaborative fields of mathematics and statistics. These fields do not require large capital investments and so do not depend on the kinds of large team projects common in some areas of physics and astronomy. Nevertheless, the collaboration implies at least an indirect relationship between institutions from both nations.

**Table 2:** Cuban scientific output in collaboration with the U.S. according to the subfield of research January 1, 1980-December 31, 2020.

Field	Subfield	Productivity	% Total in field	Mean authors
**Physics & Astronomy**	Nuclear & Particles Physics	462	63.4%	878
	Applied Physics	125	17.1%	700
	General Physics	77	10.6%	826
	Chemical Physics	25	3.4%	6
	Fluids & Plasmas	25	3.4%	75
	Astronomy & Astrophysics	8	1.1%	9
	Optics	5	0.7%	11
	Mathematical Physics	2	0.3%	4
	**Total Field**	**729**	**100%**	**767**
**Clinical Medicine**	Neurology & Neurosurgery	112	17.0%	13
	General & Internal Medicine	98	14.9%	76
	Cardiovascular System & Hematology	60	9.1%	15
	Tropical Medicine	41	6.2%	12
	Pediatrics	38	5.8%	6
	Oncology & Carcinogenesis	35	5.3%	22
	Obstetrics & Reproductive Medicine	34	5.2%	19
	Immunology	33	5.0%	12
	Nuclear Medicine & Medical Imaging	27	4.1%	11
	Pharmacology & Pharmacy	26	3.9%	9
	Arthritis & Rheumatology	24	3.6%	27
	Surgery	23	3.5%	9
	Orthopedics	14	2.1%	5
	Gastroenterology & Hepatology	10	1.5%	17
	Ophthalmology & Optometry	10	1.5%	6
	Psychiatry	10	1.5%	12
	Endocrinology & Metabolism	8	1.2%	9
	Geriatrics	8	1.2%	19
	Allergy	7	1.1%	51
	Dermatology & Venereal Diseases	7	1.1%	21
	Environmental & Occupational Health	7	1.1%	194
	Dentistry	6	0.9%	3
	Respiratory System	6	0.9%	36
	Otorhinolaryngology	4	0.6%	76
	Urology & Nephrology	4	0.6%	11
	General Clinical Medicine	3	0.5%	14
	Anesthesiology	2	0.3%	4
	Pathology	1	0.2%	15
	Sport Sciences	1	0.2%	6
	**Total field**	**659**	**100%**	**26**
**Biology**	Marine Biology & Hydrobiology	65	20.2%	5
	Zoology	58	18.1%	3
	Ecology	55	17.1%	13
	Evolutionary Biology	53	16.5%	6
	Plant Biology & Botany	44	13.7%	9
	Ornithology	29	9.0%	4
	Entomology	17	5.3%	5
	**Total field**	**321**	**100%**	**7**

Fields, and subfields are organized according to Science Metrix journal classification list.

Something similar occurs in the Clinical Medicine field. The areas Neurology & Neurosurgery, Cardiovascular system & hematology, Tropical Medicine, and General & Internal Medicine account for 47.2% of the overall productivity in this field. The mean number of authors per paper is 26. Because Cuba is a tropical country it represents a natural laboratory to develop research in tropical medicine where there has been more than a century of collaboration between U.S. and Cuban scientists going back to work on yellow fever in the late 19th century [9]. This is a strong attractor to do research in cooperation with Cuban scientists. An intriguing inference that will require further validation is that once established these cooperative relationships endure despite political obstacles.

**Fig 1:**
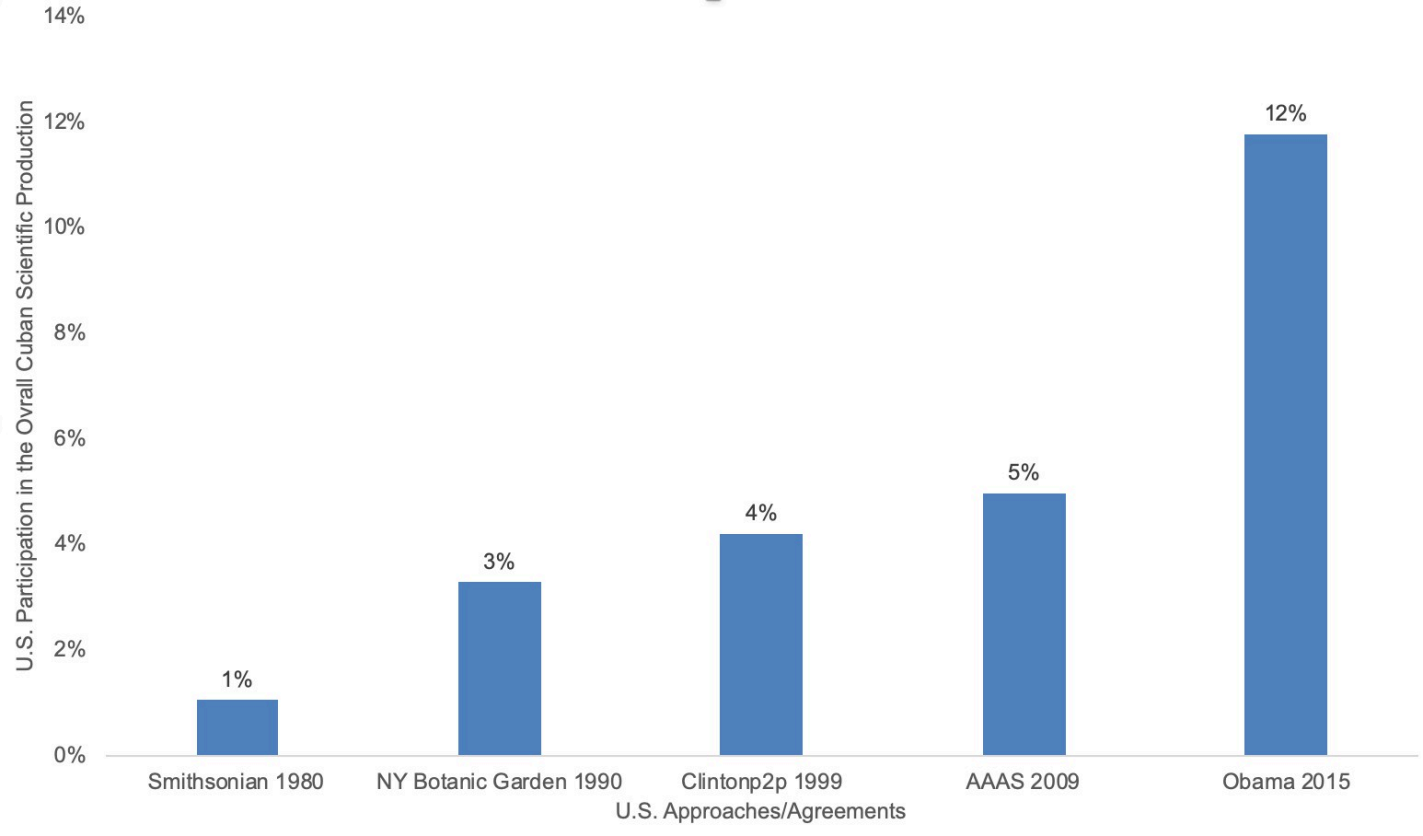
The percent of the U.S. scientist’s participation in overall Cuban scientific production. The stages are organized according to U.S. main scientific approaches/agreements. The time-line from the year of the agreement through the next significant milestone. These milestones were selected according to the documents on U.S.-Cuban scientific collaboration. Source: Clarivate Analytics Web of Science, and Scopus.

Fig 1 shows a sustained increase in the U.S. scientists’ participation in Cuban scientific productivity in the WoS and Scopus, no matter the political party in power. However, there was a significant increase with the Barack Obama 2015 approach toward normalization of diplomatic relations. During Obama’s Administration, Cuban scientific production in cooperation with U.S. scientists doubled with respect to the previous stage. Furthermore, during the eight years of the Obama Administration, Cuba published more papers in collaboration with U.S. scientists than ever before, including 52 years of the nine prior administrations. Even when the Trump administration revised the policy towards Cuba in 2017, the U.S. scientific collaboration with Cuba was maintained. As Bruce Collette of the U.S. National Marine Fisheries Service Systematics Laboratory puts it as cited by [1] “You can’t embargo science.”

### 3.2 Effects of the U.S.- Cuba scientific contacts on Cuba-U.S. joint publications

A Kruskal-Wallis (K-W) nonparametric test was conducted to test for statistically significant differences between each Cuba–U.S. scientific agreement/policy on Cuban scientific productivity because there were unequal variances in *ns* across groups. The test indicates that the U.S. agreements differed in Cuba–U.S joint scientific production *χ*^2^(4, *N* = 40) = 37.129, *p* (*Asymp*.*Sig*.) = 0.001, Tables 3 and S1.

**Table 3:** Mean rank of Cuba–U.S joint scientific productivity during each U.S. approach.

	Approach	*N*	Mean Rank
**Cuba-U.S. joint scientific production**	Smithsonian 1980	10	5.55
	NY Botanical Garden 1990	9	15.39
	Clinton p2p 1999	10	24.60
	AAAS 2009	6	31.67
	Obama 2015	6	38.50
	**Total**	**41**	

As there is no post hoc test built into the K-W test to check for mean ranks differences of the pairs of U.S. Cuba agreements or policies on Cuban productivity Post hoc Mann-Whitney tests compared the four pairs of U.S.–Cuba scientific approaches/agreements on Cuba–U.S. joint publications, using a Bonferonni corrected $p\approx 0.01\left(\frac{0.05}{4}\right)$ to indicate statistical significance S1 Table.

We compared the effects of the U.S. agreements or policies with Cuba’s Academy of Sciences on Cuba–U.S. joint productivity during the period 1980–2020 for statistical significance Table 4. The mean ranks for Cuba–U.S. joint publications during the Obama 2015 approach to the normalization of relations with Cuba (13.50, *n* = 6) was significantly higher than that of the Smithsonian–CAS 1980 agreement (5.50, *n* = 10), *z* = 3.288, *p* = 0.001, *r* = 0.83, a much larger than typical effect size difference according to [53].

**Table 4:** Comparisons of the effects of Obama’s 2015 approach with previous U.S. contacts with Cuban scientific authorities on Cuba–U.S. joint productivity.

Agreements	*Z*	*N*	*p*	*r*
**Obama 2015 Vs. Smithsonian 1980**	3.288	16	0.001	0.82*
**Obama 215 Vs. NY Botanical Garden 1990**	3.185	15	0.001	0.82*
**Obama 2015 Vs. Clinton’s p2p 1999**	3.259	16	0.001	0.81*
**Obama 2015 Vs. AAAS 2009**	2.882	12	0.004	0.83*

*p<0.01. Bonferonni corrected *p*−*value* (0.05/4) for statistical significance = 0.01. The *r* value was calculated using the conversion formula $z=\frac{z}{\sqrt{N}}$.

Also, the mean ranks for Cuba–U.S. joint publications during the Obama 2015 approach (12.50, *n* = 6) was significantly higher than that of the New York Botanical Garden–CAS 1980 agreement (5.00, *n* = 9), *z* = 3.185, *p* = 0.001, *r* = 0.82, a much larger than typical effect size difference according to [53]. The mean ranks for Cuba–U.S. joint publications during the Obama 2015 approach (13.50, *n* = 6) was significantly higher than that of Clinton’s 1999 people to people policy (5.50, *n* = 10), *z* = 3.256, *p* = 0.001, *r* = 0.81, a much larger than typical effect size difference according to [53]. The mean ranks for Cuba–U.S. joint publications during the Obama’s 2015 approach (9.50, *n* = 6) was significantly higher than that of the AAAS 2009 approach to CAS (3.50, *n* = 6), *z* = 2.882, *p* = 0.004, *r* = 0.83, a much larger than typical effect size difference according to [53].

The Obama approach toward normalization of U.S. relations with Cuba in 2015 is the one that influenced the most in the increase of Cuban scientific production in academic cooperation with the U.S. scientific institutions S2 Table. It is interesting to note that Cuban productivity rates increased steadily throughout the period investigated. Obama’s political shift in 2015 implemented specific actions such as 1) Cuban-developed medications to enter normal FDA regulatory channels [2]. 2) The U.S. Department of the Treasury granted licenses for clinical trials in the U.S. of specific Cuban medications [2]. 3) The U.S. National Institute of Health initiated a small number of relatively small grants to be administered by CRDF Global, a U.S. Non-Government Organization that implements international scientific cooperation programs [2]. The Department of Health and Human Services and the Cuban Ministry of Public Health signed a memorandum of understanding supporting cooperation and research in public health [54].

### 3.3 Cuba–U.S. scientific collaboration

**Fig 2:**
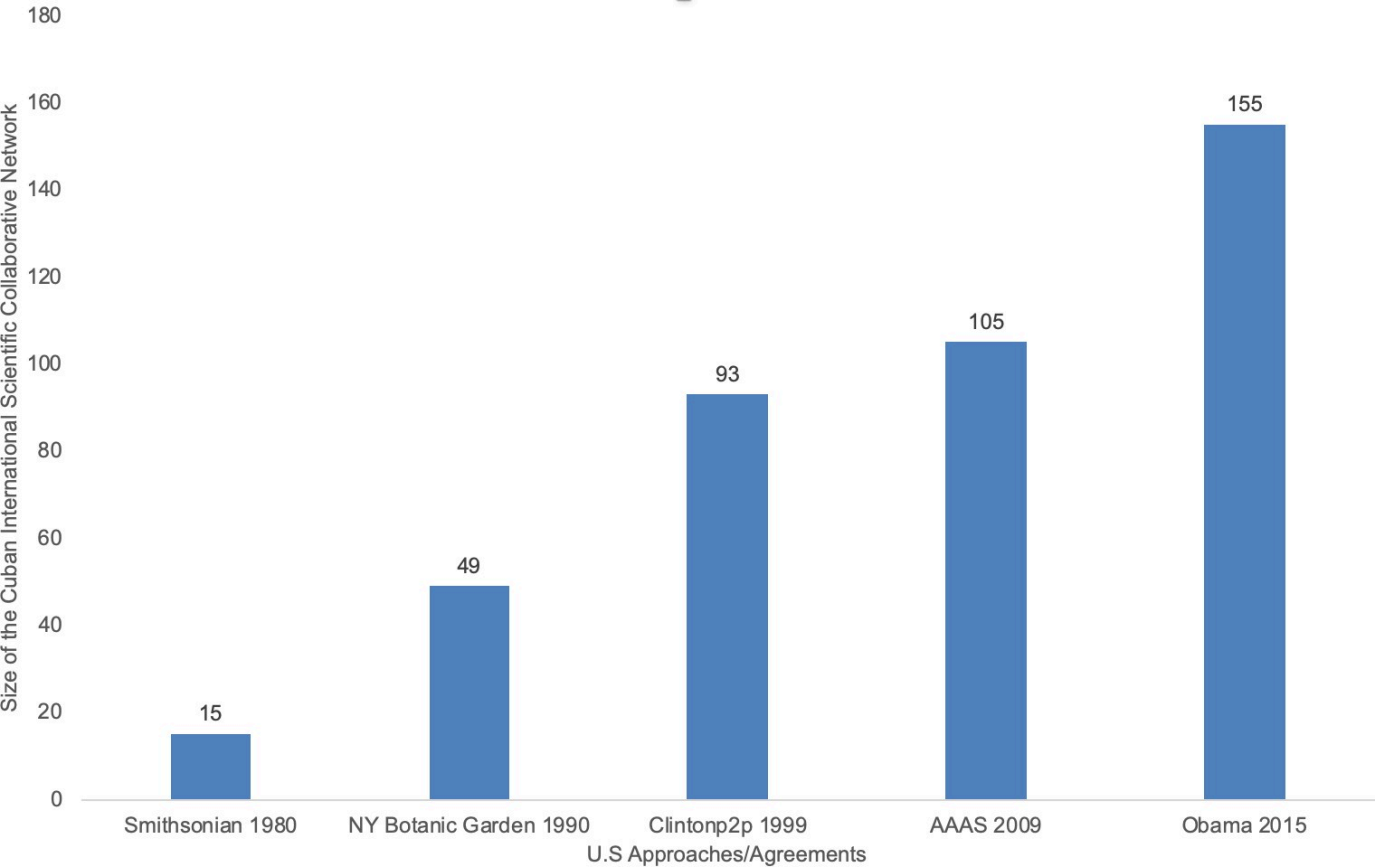
The number of countries in the Cuba–U.S. scientific international collaboration network 1980–2020. The information according to the total number of countries in the U.S.-Cuba co-authored papers in each stage.

Fig 2 shows the number of countries participating in the Cuban international scientific collaboration network. The relevance of Cuban research in the Biopharmaceutical industry, Public health care, and Meteorology may have enhanced the interest of U.S. scientists in cooperating with the Cuban scientists doing frontier research on those topics and thereby contributed to enhance the bilateral academic collaboration.

**Fig 3:**
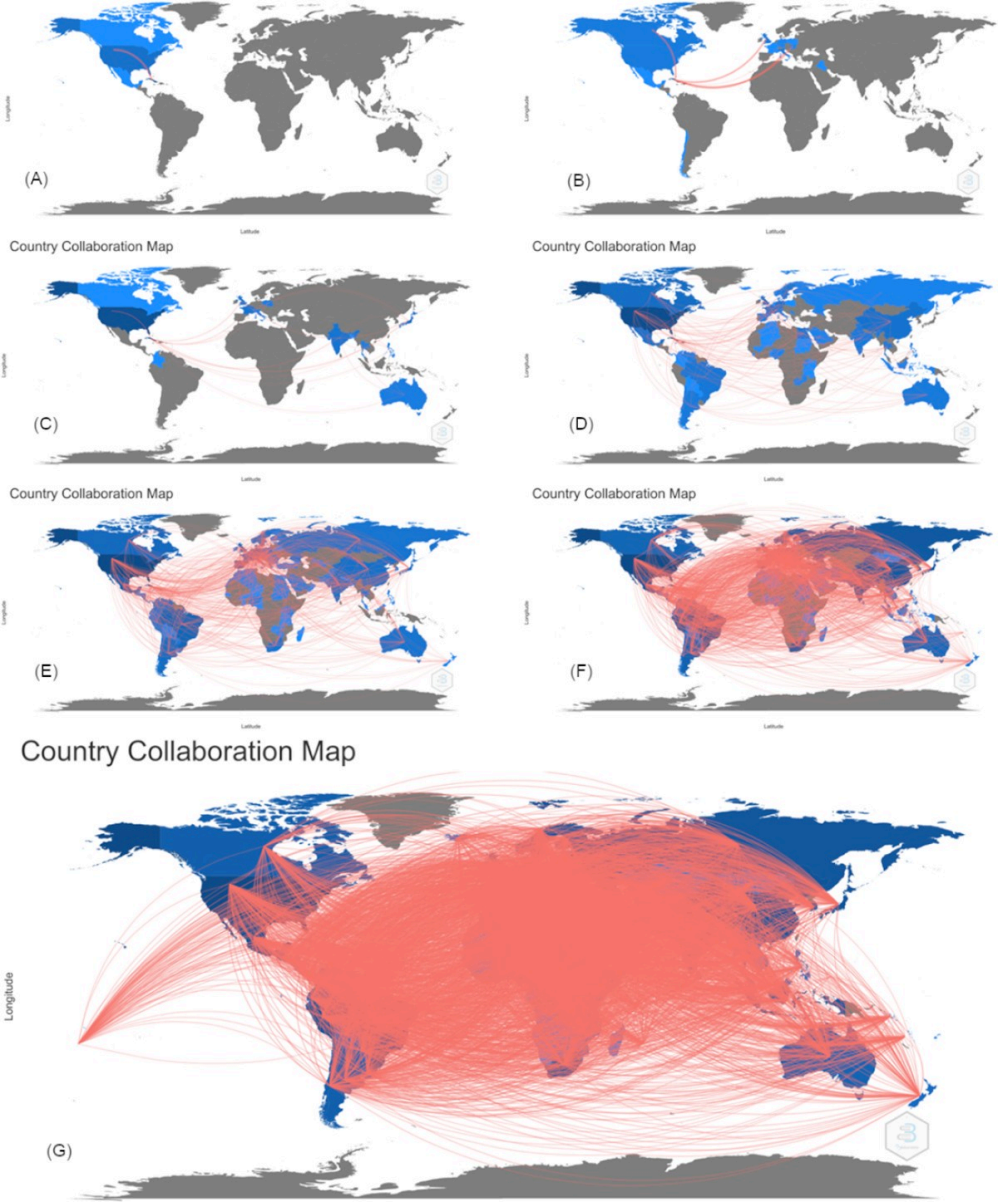
The evolution of the Cuban scientific international collaboration network 1900–2020. (A). The Cuban collaboration network 1900–1059. (B). The Cuban collaboration network 1960–1980. (C). Cuban collaboration network after the Smithsonian agreement with the Cuban Academy of Sciences. (D). Cuban collaboration network after the New York Botanical Garden agreement with the Cuban Academy of Sciences. (E). Cuban collaboration network after Bill Clinton’s p2p approach in 1999. (F). Cuban collaboration network after the visit of U.S. science leaders of the AAAS in 2009. (G). Cuban collaboration network after the Obama approach in 2015. The country collaboration maps were prepared using the R program bibliometrix, an open-source tool for quantitative research in Scientometrics. Available at: https://www.bibliometrix.org.

Fig 3 shows the dynamics of the evolution of the Cuban international scientific collaboration network. Before1960 the Cuban science system collaborated only with the U.S. Harvard University in the U.S. (Fig 3A). After the Cuban revolution in 1959 through 1979, the scientific collaboration with the U.S. froze. Cuba’s scientific collaboration with Northern America was with the University of Saskatchewan in Western Canada (Fig 3B). From 1960 to 1989, the Cuban collaboration was mainly with countries from Eastern Europe’s socialist block. After the collapse of the USSR in 1989 the internationalization and the geographical diversification enhanced Cuban collaboration with Western Europe and Latin American countries (Fig 3C). Cuba made substantial investments in certain areas of science like Biotechnology following the collapse of the USSR as a way of building the future economy. The Cuban scientific policies played a large role in stimulating foreign collaboration owing to this major strategy.

The agreement between the U.S. Smithsonian Institution and the Cuba’s Academy of Sciences in 1980 rebirth the U.S.–Cuba collaboration, which began to grow with the 1990’s agreement between New York Botanical Garden and Cuba’s Academy of Sciences. During Barack Obama’s Presidency, the number of participating countries in the Cuban scientific collaborative network doubled (Fig 3G), suggesting a growth of the Cuban scientific international links. In this stage, Cuba expanded the scientific links to 80% of countries acknowledged by the United Nations. 1980 through 2020 the density of the network grew steadily across the years.

Table 5 shows the top ten Cuban academic partners. The U.S. is ranked among the top four scientific partners from 1990 on. It is also noteworthy that Spain replaced the USSR as the most significant Cuban academic partner after the USSR collapsed in 1989. Also, Brazil and Mexico became the most important Cuban partners in Latin America. From 2010 onwards, China appears among the ten top Cuban academic partners.

**Table 5:** The top ten countries in the Cuban academic international collaborative network.

Stage	Rank									
	1	2	3	4	5	6	7	8	9	10
**Smithsonian 1980**	USSR	Ger. Dem Rep	Czechoslovakia	Italy	Hungary	France	The U.S.	Sweden	Switzerland	Spain
**NY Botanic 1990**	Spain	Mexico	U.S.	Germany	Brazil	France	Italy	Sweden	USSR	Canada
**Clinton’s p2p 1999**	Spain	Mexico	Brazil	U.S.	Germany	Italy	England	France	Belgium	Argentina
**AAAS 2009**	Spain	Mexico	Brazil	U.S.	Germany	Italy	France	England	Belgium	China
**Obama 2015**	Mexico	Brazil	Spain	U.S.	Germany	France	Italy	England	China	Switzerland

The ranking is prepared using the overall Cuban scientific output.

### 3.4 Effects of Cuban scientific collaborative network in U.S.–Cuba collaboration

We ran a power-law correlation to find out if the U.S.–Cuba scientific collaboration enhanced the growth of the Cuban international scientific collaboration network. The statistical assumptions of source population normality (Shapiro−Wilk, *p* = 0.781), the constant variance of the dependent variable in the source population regardless of the value of the independent variable (*p* = 0.05), and the independence of residuals (Durbin−Watson = 2.467) were checked and met. The scaling correlation is statistically significant *t*(1,4) = 13.91, *R*^2^ = 0.98, *p* = 0.0008 S1 Fig. To evaluate the goodness of fit of the model, we used the Predicted Residual Error Sum of Squares (*PRESS* = 0.03). Also, the results show that *α*_*SMA*_≈*α*_*OLS*_, supporting the variables are highly correlated according to a power-law.

**Fig 4:**
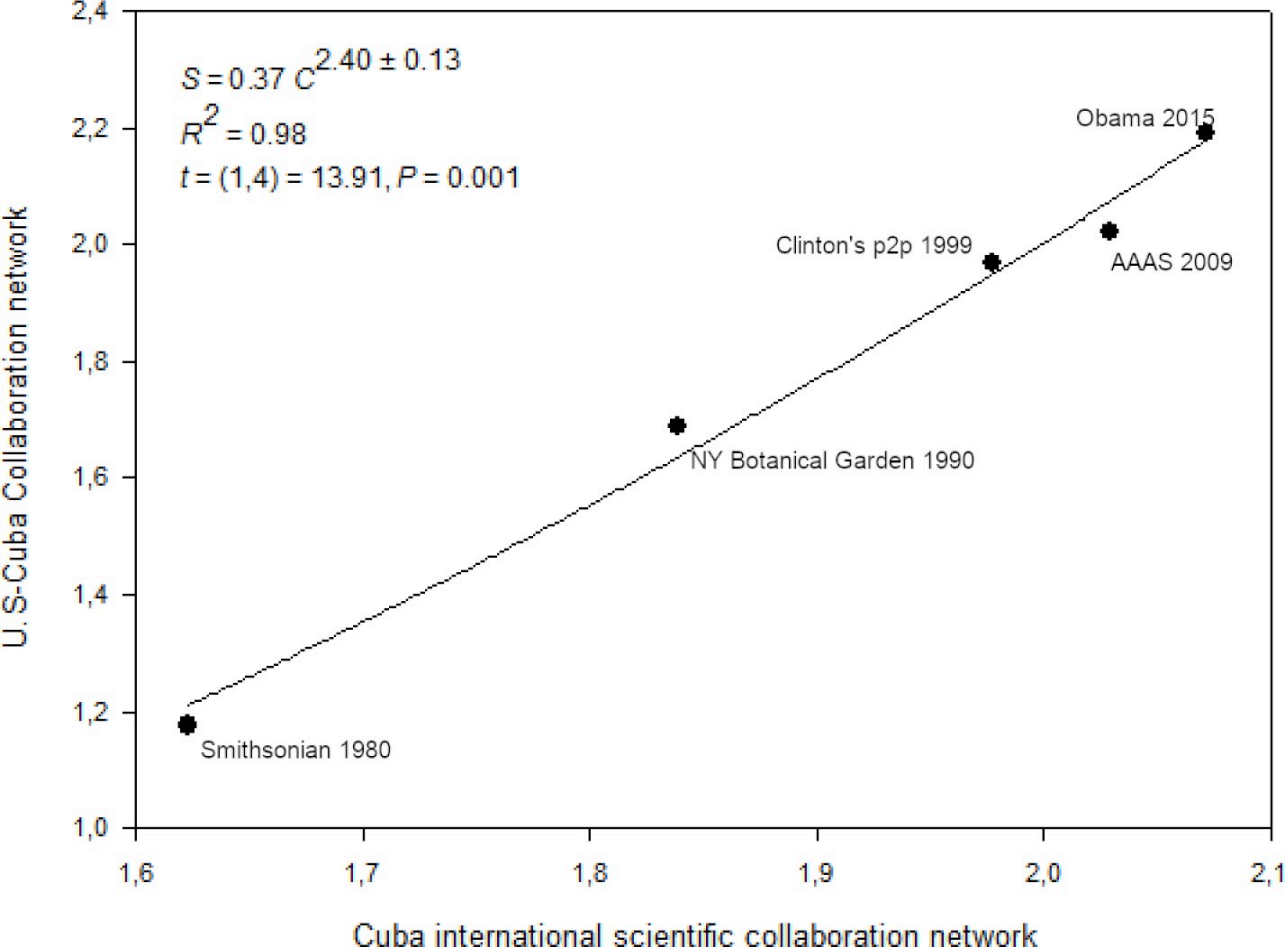
The scaling relationship between the size of the Cuban international collaboration network and the size of the Cuba–U.S. collaborative network. ***X*** = the number of countries in the overall Cuban international scientific collaboration, excluding the U.S. *Y* = the number of countries in the Cuba–U.S. collaboration network.

The result indicates there is a superliner scaling correlation between the size of the Cuban international scientific collaborative network and the growth of the Cuba–U.S. scientific collaboration, suggesting the Cuban international scientific collaborative links foster the growth of the Cuba–U.S. scientific collaboration. The Cuba–U.S. scientific collaboration grew by 2^2.40±0.13^ or 5.28 times with a doubling of the size of Cuban international scientific collaboration network Figs 4 and S1. The increase in the size of the Cuban international collaborative network indirectly enhanced collaboration with U.S scientific institutions.

In practical terms, The Cuban science system enhanced its absorptive capacities by fostering its overall international scientific collaboration network. The preferential attachment mechanism acts as an attractor to U.S. scientific institutions through the betweenness [55] of countries in the structure of the Cuban international academic collaboration network. For example, betweenness occurs when the Cuban scientists collaborate with scientists of country B and those of country B collaborate with the U.S. scientists, so Cuban scientists also collaborate with the U.S. scientists through the Cuba–B–U.S. relationship. The new countries entering the network increase the probability that Cuban scientists to collaborate with their U.S. colleagues.

## 4 Discussion

Cuban scientific productivity from 1980 through 2020 shows a sustained and steady increase. The following approaches could explain this increment.

### 4.1 The intellectual capital management

Intellectual capital is the value of an organization member’s knowledge, skills, business training, or any proprietary information that may provide the organization with a competitive advantage [56]. Intellectual capital is most commonly clustered into three categories: human capital, social or relationship capital, and structural capital. According to [57] the countries with dense social capital (highly connected) have an advantage over less connected in creating and sharing intellectual capital [58]. [59] reported that intellectual capital combined with social capital positively influences innovative capability.

The most important strength of the Cuban science system is that Cuban scientists are well trained [7] and committed to science. That is why, Cuban researchers overcome the scarceness of resources to do research. They do high-quality research in mainstream research fields with a minimum of resources by participating in international projects that involve a high number of international researchers from developed countries, including from the U.S. An important attraction is the opportunity to collaborate with high-profile scientists from the U.S. and Europe, which accelerates the transfer of new methodologies and concepts.

The Cuban Science system has highly competitive researchers in many scientific fields doing frontier research, mainly in health and natural sciences. A management framework of the Cuban Academy of Sciences with the Higher Education Ministry integrates the Higher Education institutions, Research Centers in the country’s provinces with the leading National Research Centers in Havana.

The Cuban scholars who have left Cuba could be a strength; they offer a significant opportunity because those scholars maintain their links with their colleagues on the island; they continue collaborating and serve indirectly as bridges between their hosting scientific institutions in Europe, North America, or Latin America to the scientists in Cuba. This strategy’s main weakness is that the longer the Cuban scholars remain away from home, collaboration with Cuba may decline [60]. This effect is like the strategies used by pool game players. They aim at a third ball to strike a second ball that is better positioned for the basket. Cuban scientists collaborate with scientists from third-party countries that belong to the U.S. mainstream collaborative networks. That way they also cooperate indirectly with U.S academics. This empirical evidence suggests that the growth of the Cuban international collaboration network with the U.S. is not only the consequence of a specific research policy strategy but also an emergent strategy caused by the Cuban and the U.S. scientist’s initiatives. This idea leads to the question does the scientific diaspora is a driver to foster scientific collaboration between the scientific communities of the country of origin and the hosting country?

### 4.2 The preferential attachment mechanism

The preferential attachment approach suggests that the network nodes link with a higher probability to those nodes that already have a larger number of links [61, 62]. The probability of a particular scientist acquiring new collaborators increases with the number of his or her past collaborators [50]. The U.S. science system is the node with the highest degree of centrality in the world scientific collaboration network. When the U.S. scientists collaborate with colleagues in countries that also participate in the Cuban international scientific collaborative network, the Cuban collaboration network with the U.S. academic institutions grows, through the preferential attachment mechanism. Owing to its central position, the U.S. connects to many other countries, serving as a bridge to connect Cuban scientists with other countries with semi peripheric or peripheric positions. The Cuban science system enhances its absorptive capacities by engaging in large international collaborative networks that mitigate the scarceness of resources needed to perform high-quality research in mainstream subjects. The size and density of the international collaborative network serves as an umbrella that hides the Cuba-U.S. bilateral collaboration.

The engagement of Cuban scientists in important international research projects in areas such as physics and astronomy, and clinical medicine, which are both highly networked and capital intensive, overcome legal constraints and enhances direct bilateral academic collaboration. The network embeddedness serves as an invisible bridge that facilitates the U.S. scientific collaboration with Cuban colleagues without violating restrictions imposed by the law of their homelands. This empirical evidence suggests that developing countries included in highly dense collaboration networks have a path to overcome some of the obstacles imposed by resource scarcity and also, to avoid or mitigate entry barriers raised by legal, economic, or socio-cultural limitations.

In Latin America, the Brazilian science system shows the highest degree of centrality. This country has become the most crucial scientific partner for Cuba over the past thirty years. This collaborative relationship has also enhanced the flow of new scientific knowledge and methodologies into the Cuban science system. The Cuba-Brazil academic collaboration also helps foster Cuban absorptive capacities by the preferential attachment mechanism.

It is important to also point out that there is a threshold of scientific development necessary to successfully absorb and integrate into these knowledge flows. The Cuban science system has long exceeded this threshold and is, therefore, able to take full advantage of the global knowledge ecosystem. An important issue for future work will be to better define and operationalize these knowledge thresholds and to improve our understanding of the processes of knowledge diffusion with the goal of providing a template to guide development policies in other national settings.

## 5 Conclusions

The results show that the Cuban–U.S. joint scientific production, measured as the number of articles, reviews, and proceedings paper published in the Web of Science, and Scopus increased steadily over the past forty years Tables 1 and 2, and Fig 1. The results also suggest that scientific contacts between the U.S. scientific leaders and the Cuban scientific authorities have had important accomplishments: First, the increase of cooperation between both scientific communities leading to a significant increase of joint publications S1 Table. Second, the growth of the Cuban international scientific collaboration network S1 Fig, enhancing Cuban research capabilities, and overcoming resource scarcity to thereby enabling research in mainstream subject matters.

The results confirm that scientific collaboration has been an effective diplomatic channel between Cuba and the U.S. Both scientific communities increased their collaborative efforts to solve scientific questions of primary common interest [14, 17, 18, 20, 21, 25], no matter the political party in the Oval Office or the prohibitions imposed by the U.S. Embargo on Cuba [39]. Cuba–U.S. scientists managed to turn adversaries into partners for the advancement of science [53] S1 Fig. The U.S. and Cuban scientists have proven to be an effective way to restore diplomatic relations between the two nations [16, 22, 53]. This is the most powerful message that science transmits. Science has no political color, science is committed to solving the research problems that affect humanity.

The empirical evidence presented here shows that the Obama Administration’s lowering of barriers has had the largest positive effect on Cuban collaborative scientific relationships S1 Fig, and S1 Table. With the return of Democrats to the White House in 2021, the hope of a reestablishment of the U.S. diplomatic relations with Cuba is back. The results suggest that the Cuban–U.S. collaboration networks are resilient to restrictive policies [1, 15]. But a particularly important question for the future is how robust is the collaboration system in the face of greater political restrictions?

The use of the general license (www.treasury.gov/resource-center/sanctions/Programs/pages/cuba.aspx) in effect which includes academics, allows U.S. university scientists, to travel to Cuban scientific institutions, if they work on academic pursuits during the visit, with the intention to produce scientific publications. This would significantly increase the development of research in joint and mutually beneficial scientific interests [7, 9, 10, 12, 39]. The use of the U.S. Treasury Department would help for transferring scientific equipment to Cuba. Using this license, it was possible the installation of a global positioning system (GPS) receiver to measure atmospheric water vapor at the *Grupo de Óptica Atmosférica de Camagüey* (GOAC) at the Camagüey Meteorological Center in Camagüey, Cuba [7, 10].

[10] summarized the opportunities of the above-mentioned license from the U.S. Treasury Department: “E. Full-time professionals conducting professional research or attending certain professional meetings “1. Professional research. Full-time professionals are authorized to engage in Cuba travel-related transactions and such additional transactions that are directly incident to conducting professional research in their professional areas pursuant to § 515.564(a)(1) of the Regulations, provided that their research (1) is of a noncommercial academic nature; (2) comprises a full work schedule in Cuba; (3) has a substantial likelihood of public dissemination; and (4) does not fall within certain categories listed in § 515.564(c)-(e).”

## Supporting information

S1 Fig The scaling relationship between the size of the Cuban international collaboration network and the size of the Cuba-U.S. collaborative network. (PDF)

S1 Table Mean rank of Cuba-U.S joint scientific productivity during each U.S. approach. (PDF)

S2 Table Comparisons of the Obama’s 2015 with previous U.S. approaches with Cuba on Cuba-U.S. joint productivity. (PDF)

## References

[1] DeWeerdt S. Embargoing science: US policy toward Cuba and scientific collaboration. Bioscience. 2001;51(8):612–. doi: 10.1641/0006-3568(2001)051[0612:Esuptc]2.0.Co;2 WOS:000170683500005.

[2] Pastrana SJ, Gual-Soler M, Wang TC. Promoting Scientific Cooperation in Times of Diplomatic Challenges: Sustained Partnership between the Cuban Academy of Sciences and the American Association for the Advancement of Science. MEDICC. 2018;20(2):23–6. 10.37757/MR2018.V20.N2.5 29773772

[3] Pérez-Riverol Y. Trends in Cuban research output: publications and patents. [Manuscript]. In press 2000.

[4] Lempinen EW. U.S. Science Delegation Completes Hopeful Three-Day Visit to Cuba https://www.aaas.org/news/us-science-delegation-completes-hopeful-three-day-visit-cuba: AAAs.org; 2009 [cited 2021 April 15 2021]. Available from: https://www.aaas.org/news/us-science-delegation-completes-hopeful-three-day-visit-cuba.

[5] Khemlani A. World Health Organization. Cuba: WHO praises efforts and contributions to health education and prevention https://www.latinpost.com/articles/17618/20140720/who-praises-cuban-efforts-and-contributions-to-health-education-and-prevention.htm: Latin Post; 2014 [cited 2021 April 18 2021]. Available from: https://www.latinpost.com/articles/17618/20140720/who-praises-cuban-efforts-and-contributions-to-health-education-and-prevention.htm.

[6] Abreu MT, Damas OM, Pinol Jimenez FN, Canete Villafranca R. United States-Cuba Research Collaborations: Opening Bridges for Gastroenterology. Gastroenterology. 2017;152(6):1267–9. Epub 2017/03/23. doi: 10.1053/j.gastro.2017.03.011 . 28327366

[7] Abreu MT, Damas OM, Jimenez FNP, Villafranca RC. United States-Cuba Research Collaborations: Opening Bridges for Gastroenterology. Gastroenterology. 2017;152(6):1267–9. doi: 10.1053/j.gastro.2017.03.011 WOS:000401811300012. 28327366

[8] Mega-Rodríguez E. Can Cuba beat COVID with its homegrown vaccines? Nature. 2021. doi: 10.1038/d41586-021-01126-4 33927405

[9] Pastrana SJ, Clegg MT. U.S.-Cuban scientific relations. Science. 2008;322(5900):345. doi: 10.1126/science.1162561 . 18927359

[10] Anthes R, Robock A, Antuña-Marrero JC, García O, Braun JJ, Arredondo RE. Cooperation on GPS Meteorology between the United States and Cuba. Bulletin of the American Meteorological Society. 2015;96(7):1079–88. doi: 10.1175/bams-d-14-00171.1

[11] Lempinen EW. Oceans, Weather, Health—U.S. Researchers Explore Potential Collaboration with Cuban Colleagues https://www.aaas.org/news/oceans-weather-health-us-researchers-explore-potential-collaboration-cuban-colleagues: AAAS.org; 2011 [cited 2021 April 15 2021]. Available from: https://www.aaas.org/news/oceans-weather-health-us-researchers-explore-potential-collaboration-cuban-colleagues.

[12] Heger M. Delegation paves way for US-Cuba research collaborations. Nature Medicine. 2016;22(6):569–. doi: 10.1038/nm0616-569 WOS:000377476000001. 27270767

[13] Demain AL. Scientific links with Cuba flourish despite US embargo. Nature. 2009;457:1079. 10.1038/4571079c 19242451

[14] Carbonell R, Hill S. Opportunities for US/Cuba collaborations in biopharmaceutical development and manufacturing. Abstracts of Papers of the American Chemical Society. 2015;250. WOS:000432475700238.

[15] Canete R, Goodman KW. Cuba-US collaboration and the role of bioethics. Lancet. 2015;385(9972):945-. doi: 10.1016/S0140-6736(15)60523-2 WOS:000350886900023. 25784342

[16] Wren K. Science Diplomacy, Visit to Cuba Produces Historic Agreement https://www.aaas.org/news/science-diplomacy-visit-cuba-produces-historic-agreement: AAAS News; 2014 [cited 2021 April 15 2021]. Available from: https://www.aaas.org/news/science-diplomacy-visit-cuba-produces-historic-agreement.

[17] Machlis G, Frankovich TA, Alcolado PM, Garcia-Machado E, Hernandez-Zanuy AC, Hueter RE, et al. OCEAN POLICY US-Cuba Scientific Collaboration Emerging Issues and Opportunities in Marine and Related Environmental Sciences. Oceanography. 2012;25(2):227–31. doi: 10.5670/oceanog.2012.63 WOS:000306162100029.

[18] Chapman HJ, Armas-Perez LA, Lauzardo M, Gonzalez-Ochoa ER. Moving Closer to Tuberculosis Elimination through Institutional Scientific Collaboration: Opportunities for Cuba and the USA. Medicc Review. 2018;20(2):59–63. WOS:000434311900013. 29773780 10.37757/MR2018.V20.N2.14

[19] Echegoyen L. US-Cuba collaborations in chemical research: Fact or fantasy? Abstracts of Papers of the American Chemical Society. 2002;223:U168–U. WOS:000176296700778.

[20] Scott W, Fuller A, Dounay A, Samaritoni J, Odonnell M, Dave P, et al. Ernest Eliel Workshop: US and Cuba collaboration in chemistry education and neglected disease drug discovery. Abstracts of Papers of the American Chemical Society. 2018;255. WOS:000435537703452.

[21] Wang LD. ACS explores potential collaborations with Cuba. Chemical & Engineering News. 2016;94(29):35–. WOS:000380581200046.

[22] Fink GR, Leshner AI, Turekian VC. Science diplomacy with Cuba. Science. 2014;344(6188):1065. doi: 10.1126/science.1256312 . 24904126

[23] Ronda-Pupo GA, Katz JS. The scaling relationship between citation-based performance and international collaboration of Cuban articles in natural sciences. Scientometrics. 2016;107(3):1423–34. doi: 10.1007/s11192-016-1939-9

[24] Pastrana SJ. Science in U.S.-Cuba relations. Science. 2015;348(6236):735. doi: 10.1126/science.aaa9542 25977525

[25] Canete R, Goodman KW. Cuba-US Collaboration: The Pandemic Imperative. Medicc Review. 2021;23(1):89–. doi: 10.37757/MR2021.V23.N1.3 WOS:000614256600015. 33780428

[26] Aria M, Cuccurullo C. bibliometrix: An R-tool for comprehensive science mapping analysis. Journal of Informetrics. 2017;11(4):959–75. doi: 10.1016/j.joi.2017.08.007

[27] Galban-Rodriguez E, Torres-Ponjuan D, Marti-Lahera Y, Arencibia-Jorge R. Measuring the Cuban scientific output in scholarly journals through a comprehensive coverage approach. Scientometrics. 2019;121(2):1019–43. doi: 10.1007/s11192-019-03233-6 WOS:000490369400003.

[28] Arencibia-Jorge R, Corera-Alvarez E, Chinchilla-Rodriguez Z, de Moya-Anegon F. Scientific output of the emerging Cuban biopharmaceutical industry: a scientometric approach. Scientometrics. 2016;108(3):1621–36. doi: 10.1007/s11192-016-2023-1 WOS:000382914200032.

[29] Katz JS, Martin BR. What is research collaboration? Research Policy. 1997;26(1):1–18. doi: 10.1016/S0048-7333(96)00917-1

[30] Beaver DB, Rosen R. Studies in scientific collaboration. Scientometrics. 1979;1(2):133–49. doi: 10.1007/bf02016966

[31] Beaver DD. Reflections on Scientific Collaboration (and its Study): Past, Present, and Future. 2000.

[32] Kahn M. Co-authorship as a proxy for collaboration: a cautionary tale. Sci Publ Policy. 2018;45(1):117–23. doi: 10.1093/scipol/scx052 WOS:000427377700011.

[33] Katz JS, Hicks D. How much is a collaboration worth? A calibrated bibliometric model. Scientometrics. 1997;40(3):541–54. doi: 10.1007/BF02459299

[34] Leydesdorff L, Wagner C. International Collaboration in Science and the Formation of a Core Group. Journal of Informetrics. 2008;2(4):317–25. doi: 10.1016/j.joi.2008.07.003

[35] Lee S. The Impact of Research Collaboration on Scientific Productivity. Social Studies of Science. 2005;35(5):673–702. doi: 10.1177/0306312705052359

[36] Society TR. Knowledge, networks and nations Global scientific collaboration in the 21st century. 2013.

[37] Narvaez-Berthelemot N. An index to measure the international collaboration of developing countries based on the participation of national institutions: The case of Latin America. Scientometrics. 1995;34(1):37–44. doi: 10.1007/bf02019171

[38] Ronda-Pupo GA, Katz JS. The Scaling Relationship between Citation-Based Performance and Scientific Collaboration in Natural Sciences. Journal of the Association for Information Science and Technology. 2017;68(5):1257–65. doi: 10.1002/asi.23759

[39] Hogue C. Door opened to US-Cuba collaboration. Chemical & Engineering News. 2016;94(46):19–. WOS:000388551900045.

[40] Chapman HJ, Armas-Perez LA, Lauzardo M, Gonzalez-Ochoa ER. Moving Closer to Tuberculosis Elimination through Institutional Scientific Collaboration: Opportunities for Cuba and the USA (vol 20, pg 59, 2018). Medicc Review. 2018;20(3):9–. WOS:000514167800004. 10.37757/MR2018.V20.N2.14 29773780

[41] Leech NL, Barret KC, Morgan GA. IBM SPSS for Intermediate Statistics. Use and Interpretation. 4 ed. New York: Routledge Taylor & Francis Group; 2011. 300 p.

[42] Katz JS. What Is a Complex Innovation System? Plos One. 2016;11(6):e0156150. doi: 10.1371/journal.pone.0156150 ; PubMed Central PMCID: PMC4892634. 27258040 PMC4892634

[43] Ronda-Pupo GA. The citation-based impact of complex innovation systems scales with the size of the system. Scientometrics. 2017;112(1):141–51. doi: 10.1007/s11192-017-2401-3

[44] Marquardt DW. An Algorithm for Least Squares Estimation of Parameters. Journal of the Society of Industrial and Applied Mathematics. 1963;11:431–41.

[45] Katz JS. Scale-independent bibliometric indicators. Measurement. 2005;3(1):24–8. doi: 10.1207/s15366359mea0301_3

[46] Katz JS. The european innovation system: A scale-independent model http://www.sussex.ac.uk/Users/sylvank/plm: University of Sussex; 2003 [cited 2014 November 28]. Available from: http://www.sussex.ac.uk/Users/sylvank/plm.

[47] Huxley JS. Problems of Relative Growth. London: Methuen & Co. LTD; 1923. doi: 10.1126/science.58.1502.291

[48] Katz JS, Cothey V. Web indicators for complex innovation systems. Research Evaluation. 2006;15(2):85–95. doi: 10.3152/147154406781775922

[49] West GB. Scale. New York: Penguin Press; 2017.

[50] Newman ME. Clustering and preferential attachment in growing networks. Phys Rev E Stat Nonlin Soft Matter Phys. 2001;64(2 Pt 2):025102. doi: 10.1103/PhysRevE.64.025102 . 11497639

[51] Leguendre P, Leguendre L. Numerical Ecology. 3 ed. Modelong DiE, editor. Great Britain: Elsevier B. V; 2012 2012.

[52] Guzman-Sanchez MV, Pinon-Lora M, Villasenor-Garcia EA, Jimenez-Andrade JL, Carrillo-Calvet H. Characterization of the Cuban biopharmaceutical industry from collaborative networks. Scientometrics. 2018;115(3):1533–48. doi: 10.1007/s11192-018-2719-5 WOS:000437262800024.

[53] Cohen J. Statistical Power and Analysis for the Behavioral Sciences. 2 ed. New Jersey: Lawrence Erlbaum; 1988.

[54] Keck CW. The United States and Cuba—Turning Enemies into Partners for Health. N Engl J Med. 2016;375(16):1507–9. Epub 2016/11/01. doi: 10.1056/NEJMp1608859 . 27797312

[55] Freeman LC, Borgatti S P, White DR. Centrality in valued graphs: A measure of betweenness based on network flow. Social Networks. 1991;13:141–54.

[56] Roos J. Exploring the concept of intellectual capital (IC). Long Range Planning. 1998;31(1):150–3. doi: 10.1016/s0024-6301(97)87431-6

[57] Nahapiet J, Ghoshal S. Social Capital, Intellectual Capital, and the Organizational Advantage. The Academy of Management Review. 1998;23(2). doi: 10.2307/259373

[58] Nguyen DQ. The impact of intellectual capital and knowledge flows on incremental and radical innovation. Asia-Pacific Journal of Business Administration. 2018;10(2/3):149–70. doi: 10.1108/apjba-03-2018-0044

[59] Subramaniam M, Youndt MA. The Influence of Intellectual Capital on the Types of Innovative Capabilities. Academy of Management Journal. 2005;48(3):450–63. doi: 10.5465/amj.2005.17407911

[60] Palacios-Callender M, Roberts SA. Scientific collaboration of Cuban researchers working in Europe: understanding relations between origin and destination countries. Scientometrics. 2018;117(2):745–69. doi: 10.1007/s11192-018-2888-2 WOS:000451754300005. 30595611 PMC6280978

[61] Barabási AL, Albert R. Emergence of Scaling in Random Networks. Science. 1999;286(5439):509–12. doi: 10.1126/science.286.5439.509 10521342

[62] Barabási AL, Jeong H, Néda Z, Ravasz E, Schubert A, Vicsek T. Evolution of the social network of scientific collaborations. Physica A: Statistical Mechanics and its Applications. 2002;311(3–4):590–614. doi: 10.1016/s0378-4371(02)00736-7

