# Supplementary material for: Cuba—U.S. scientific collaboration: Beyond the embargo
Source: PLoS One. 2021 Jul 22;16(7):e0255106. doi: 10.1371/journal.pone.0255106 (PMC8297818; doi:10.1371/journal.pone.0255106)
Supplement: S1 Fig — (PDF) [file pone.0255106.s001.pdf]

Nonlinear Regression **lunes, mayo 03, 2021, 14:21:01**

Data Source: Data 1 in PLoS
Equation: Power; 2 Parameter
f = a*x^b

**R**	**Rsqr**	**Adj Rsqr**	**Standard Error of Estimate**
0,9940	0,9881	0,9842	0,0499

	**Coefficient**	**Std. Error**	**t**	**P**
a	0,3768	0,0442	8,5331	0,0034
b	2,4096	0,1732	13,9121	0,0008

Analysis of Variance:

	**DF**	**SS**	**MS**
Regression	2	16,9902	8,4951
Residual	3	0,0075	0,0025
Total	5	16,9976	3,3995

Corrected for the mean of the observations:

	**DF**	**SS**	**MS**	**F**	**P**
Regression	1	0,6231	0,6231	249,8146	0,0006
Residual	3	0,0075	0,0025		
Total	4	0,6306	0,1576		

Statistical Tests:

PRESS 0,0275

Durbin-Watson Statistic 2,4679 Passed

Normality Test (Shapiro-Wilk) Passed (P = 0,7818)

W Statistic= 0,9563 Significance Level = 0,0500

Constant Variance Test Passed (P = 0,0500)

Power of performed test with alpha = 0,0500: 0,9843

Influence Diagnostics:

Row	Cook's Dist
1	2,0922
2	0,3452
3	0,0295
4	0,3728
5	0,0334

Fit Equation Description:

```
[Variables]
x = col(1)
y = col(2)
reciprocal_y = 1/abs(y)
reciprocal_ysquare = 1/y^2
reciprocal_pred = 1/abs(f)
reciprocal_predsqr = 1/f^2
[Parameters]
a = mean(y) "Auto {{previous: 0,376809}}
b = 1 "Auto {{previous: 2,40962}}
[Equation]
f = a*x^b
fit f to y
"fit f to y with weight reciprocal_y
"fit f to y with weight reciprocal_ysquare
```

"fit f to y with weight reciprocal_pred
"fit f to y with weight reciprocal_predsqr
[Constraints]
b>0
[Options]
tolerance=0,0000000001
stepsize=1
iterations=200

Number of Iterations Performed = 11
