# Supplementary material for: Cuba—U.S. scientific collaboration: Beyond the embargo
Source: PLoS One. 2021 Jul 22;16(7):e0255106. doi: 10.1371/journal.pone.0255106 (PMC8297818; doi:10.1371/journal.pone.0255106)
Supplement: S1 Table — (PDF) [file pone.0255106.s002.pdf]

```
 GET
  FILE='/Users/Guillermo/Desktop/PLoS.sav'.
DATASET NAME DataSet1 WINDOW=FRONT.
NPAR TESTS
  /K-W=USCuba BY USApproach(0 4)
  /MISSING ANALYSIS.
```

NPar Tests

Notes

Output Created		03-MAY-2021 15:53:20
Comments		
Input	Data	/Users/Guillermo/Desktop/PLoS.sav
	Active Dataset	DataSet1
	Filter	<none>
	Weight	<none>
	Split File	<none>
	N of Rows in Working Data File	41
Missing Value Handling	Definition of Missing	User-defined missing values are treated as missing.
	Cases Used	Statistics for each test are based on all cases with valid data for the variable(s) used in that test.
Syntax		NPAR TESTS /K-W=USCuba BY USApproach(0 4) /MISSING ANALYSIS.
Resources	Processor Time	00:00:00,00
	Elapsed Time	00:00:00,00
	Number of Cases Allowed[a]	112347

a. Based on availability of workspace memory.

```
[DataSet1] /Users/Guillermo/Desktop/PLoS.sav
```

Kruskal-Wallis Test

Ranks

	USApproach	N	Mean Rank
USCuba	Smithsonian 1980	10	5,55
	NY Botanical Garden 1990	9	15,39
	Clinton p2p 1999	10	24,60
	AAAS 2009	6	31,67
	Obama 2015	6	38,50
	Total	41	

Test Statistics[a,b]

	USCuba
Chi-Square	37,129
df	4
Asymp. Sig.	,000

a. Kruskal Wallis Test

b. Grouping Variable: USApproach
