# Supplementary material for: Cuba—U.S. scientific collaboration: Beyond the embargo
Source: PLoS One. 2021 Jul 22;16(7):e0255106. doi: 10.1371/journal.pone.0255106 (PMC8297818; doi:10.1371/journal.pone.0255106)
Supplement: S2 Table — (PDF) [file pone.0255106.s003.pdf]

```
NPAR TESTS
  /M-W= USCuba BY USApproach(4 0)
  /MISSING ANALYSIS.
```

NPar Tests

Notes

Output Created		03-MAY-2021 15:55:18
Comments		
Input	Data	/Users/Guillermo/Desktop/PLoS.sav
	Active Dataset	DataSet1
	Filter	<none>
	Weight	<none>
	Split File	<none>
	N of Rows in Working Data File	41
Missing Value Handling	Definition of Missing	User-defined missing values are treated as missing.
	Cases Used	Statistics for each test are based on all cases with valid data for the variable(s) used in that test.
Syntax		NPAR TESTS /M-W= USCuba BY USApproach(4 0) /MISSING ANALYSIS.
Resources	Processor Time	00:00:00,00
	Elapsed Time	00:00:00,00
	Number of Cases Allowed[a]	112347

a. Based on availability of workspace memory.

```
[DataSet1] /Users/Guillermo/Desktop/PLoS.sav
```

Mann-Whitney Test

Ranks

	USApproach	N	Mean Rank	Sum of Ranks
USCuba	Smithsonian 1980	10	5,50	55,00
	Obama 2015	6	13,50	81,00
	Total	16		

Test Statistics[a]

	USCuba
Mann-Whitney U	,000
Wilcoxon W	55,000
Z	-3,288
Asymp. Sig. (2-tailed)	,001
Exact Sig. [2*(1-tailed Sig.)]	,000[b]

a. Grouping Variable: USApproach

b. Not corrected for ties.

```
NPAR TESTS
 /M-W= USCuba BY USApproach(4 1)
 /MISSING ANALYSIS.
```

NPar Tests

Notes

Output Created		03-MAY-2021 15:55:33
Comments		
Input	Data	/Users/Guillermo/Desktop/PLoS.sav
	Active Dataset	DataSet1
	Filter	<none>
	Weight	<none>
	Split File	<none>
	N of Rows in Working Data File	41
Missing Value Handling	Definition of Missing	User-defined missing values are treated as missing.
	Cases Used	Statistics for each test are based on all cases with valid data for the variable(s) used in that test.
Syntax		NPAR TESTS /M-W= USCuba BY USApproach(4 1) /MISSING ANALYSIS.
Resources	Processor Time	00:00:00,00
	Elapsed Time	00:00:00,00
	Number of Cases Allowed[a]	112347

a. Based on availability of workspace memory.

```
[DataSet1] /Users/Guillermo/Desktop/PLoS.sav
```

Mann-Whitney Test

Ranks

	USApproach	N	Mean Rank	Sum of Ranks
USCuba	NY Botanical Garden 1990	9	5,00	45,00
	Obama 2015	6	12,50	75,00
	Total	15		

Test Statistics[a]

	USCuba
Mann-Whitney U	,000
Wilcoxon W	45,000
Z	-3,185
Asymp. Sig. (2-tailed)	,001
Exact Sig. [2*(1-tailed Sig.)]	,000[b]

a. Grouping Variable: USApproach

b. Not corrected for ties.

```
NPAR TESTS
 /M-W= USCuba BY USApproach(4 2)
 /MISSING ANALYSIS.
```

NPar Tests

Notes

Output Created		03-MAY-2021 15:55:46
Comments		
Input	Data	/Users/Guillermo/Desktop/PLoS.sav
	Active Dataset	DataSet1
	Filter	<none>
	Weight	<none>
	Split File	<none>
	N of Rows in Working Data File	41
Missing Value Handling	Definition of Missing	User-defined missing values are treated as missing.
	Cases Used	Statistics for each test are based on all cases with valid data for the variable(s) used in that test.
Syntax		NPAR TESTS /M-W= USCuba BY USApproach(4 2) /MISSING ANALYSIS.
Resources	Processor Time	00:00:00,00
	Elapsed Time	00:00:00,00
	Number of Cases Allowed[a]	112347

a. Based on availability of workspace memory.

```
[DataSet1] /Users/Guillermo/Desktop/PLoS.sav
```

Mann-Whitney Test

Ranks

	USApproach	N	Mean Rank	Sum of Ranks
USCuba	Clinton p2p 1999	10	5,50	55,00
	Obama 2015	6	13,50	81,00
	Total	16		

Test Statistics[a]

	USCuba
Mann-Whitney U	,000
Wilcoxon W	55,000
Z	-3,256
Asymp. Sig. (2-tailed)	,001
Exact Sig. [2*(1-tailed Sig.)]	,000[b]

a. Grouping Variable: USApproach

b. Not corrected for ties.

```
NPAR TESTS
 /M-W= USCuba BY USApproach(4 3)
 /MISSING ANALYSIS.
```

NPar Tests

Notes

Output Created		03-MAY-2021 15:55:58
Comments		
Input	Data	/Users/Guillermo/Desktop/PLoS.sav
	Active Dataset	DataSet1
	Filter	<none>
	Weight	<none>
	Split File	<none>
	N of Rows in Working Data File	41
Missing Value Handling	Definition of Missing	User-defined missing values are treated as missing.
	Cases Used	Statistics for each test are based on all cases with valid data for the variable(s) used in that test.
Syntax		NPAR TESTS<br>/M-W= USCuba BY USApproach(4 3)<br>/MISSING ANALYSIS.
Resources	Processor Time	00:00:00,00
	Elapsed Time	00:00:00,00
	Number of Cases Allowed[a]	112347

a. Based on availability of workspace memory.

```
[DataSet1] /Users/Guillermo/Desktop/PLoS.sav
```

Mann-Whitney Test

Ranks

	USApproach	N	Mean Rank	Sum of Ranks
USCuba	AAAS 2009	6	3,50	21,00
	Obama 2015	6	9,50	57,00
	Total	12		

Test Statistics[a]

	USCuba
Mann-Whitney U	,000
Wilcoxon W	21,000
Z	-2,882
Asymp. Sig. (2-tailed)	,004
Exact Sig. [2*(1-tailed Sig.)]	,002[b]

a. Grouping Variable: USApproach

b. Not corrected for ties.
